# Response of microbial community of organic-matter-impoverished arable soil to long-term application of soil conditioner derived from dynamic rapid fermentation of food waste

**DOI:** 10.1371/journal.pone.0175715

**Published:** 2017-04-18

**Authors:** Jiaqi Hou, Mingxiao Li, Xuhui Mao, Yan Hao, Jie Ding, Dongming Liu, Beidou Xi, Hongliang Liu

**Affiliations:** 1 State Key Laboratory of Water Environment Simulation, School of Environment, Beijing Normal University, Beijing, China; 2 State Key Laboratory of Environmental Criteria and Risk Assessment, Chinese Research Academy of Environmental Sciences, Beijing, China; 3 School of Resource and Environmental Science, Wuhan University, Wuhan, China; Natural Environment Research Council, UNITED KINGDOM

## Abstract

Rapid fermentation of food waste can be used to prepare soil conditioner. This process consumes less time and is more cost-effective than traditional preparation technology. However, the succession of the soil microbial community structure after long-term application of rapid fermentation-derived soil conditioners remains unclear. Herein, dynamic rapid fermentation (DRF) of food waste was performed to develop a soil conditioner and the successions and diversity of bacterial communities in an organic-matter-impoverished arable soil after six years of application of DRF-derived soil conditioner were investigated. Results showed that the treatment increased soil organic matter (SOM) accumulation and strawberry yield by 5.3 g/kg and 555.91 kg/ha, respectively. Proteobacteria, Actinobacteria, Acidobacteria, and Firmicutes became the dominant phyla, occupying 65.95%–77.52% of the bacterial sequences. Principal component analysis (PCA) results showed that the soil bacterial communities were largely influenced by the treatment. Redundancy analysis (RDA) results showed that the relative abundances of Gemmatimonadetes, Chloroflexi, Verrucomicrobia, Nitrospirae, and Firmicutes were significantly correlated with soil TC, TN, TP, NH_4_^+^-N, NO_3_^−^-N, OM, and moisture. These communities were all distributed in the soil samples collected in the sixth year of application. Long-term treatment did not enhance the diversity of bacterial species but significantly altered the distribution of major functional bacterial communities in the soils. Application of DRF-derived soil conditioner could improve the soil quality and optimize the microbial community, ultimately enhancing fruit yields.

## Introduction

The increasing population and food demand in the past decades have resulted in unprecedented challenges for agriculture [1–2]. Extensive rehabilitation and additional nutrients were added into soils to remedy these problems. However, such activities worsened the qualities of arable soil and led to widespreadorganic-matter (OM)-impoverished soils. Rates of crop yield decreased and even became stagnant in many countries probably caused by the loss of soil organic carbon that was not timely feedback in the arable soils [3]. As food waste always contains substantial amounts of organic matters and plant nutrients mainly originate from agriculture soil, it is often recognized as an ideal raw material for preparation of organic fertilizer used to complement soil organic carbon [4–5]. However, traditional composting of food waste is sometimes not technically or economically feasible [6]. This process often requires one or two months to ensure sufficient humification, because food waste usually has high moisture (60%–70%) and high fat content (25%–40%). Food waste tends to release large amounts of secondary pollution, such as leachates and odor pollutants [7], during composting. Moreover, the large occupied area for composting also leads to challenges in soil services and land management. Thus, a rapid fermentation technology is proposed to prepare soil fertilizer/conditioner with high organic carbon.

Fertilization using soil fertilizer/conditioner can strongly influence the soil physicochemical properties and improve soil fertility by managing soil nutrients and regulating the carbon or nitrogen cycle [8–10]. Fertilization also has an impact on the microbial link between soil carbon or nitrogen mineralization or humification [11–13]. Soil microbial communities considerably affect the dynamics of SOM and soil function through enzymes and their contributions to nutrient cycling [14–15]. However, less than 10% of microorganisms can be cultivated, resulting in significantly undiscovered microbial diversity and function [16]. Numerous molecular biological approaches have been applied in recent years to determine the changes and succession in soil bacterial communities. Several approaches use phospholipid fatty acid and denaturing gradient gel electrophoresis based on 16S rRNA genes. These techniques can identify the specific microorganism which may be meaningful for a certain study [17]. However, these approaches are also time-consuming and may introduce an incomplete estimate of phylogenetic diversity, particularly in environmental samples containing diverse microorganisms [18]. By contrast, high-throughput sequencing can provide a large number of sequences to elucidate the entire profile of microbial communities [19]. In addition, this process facilitates the analysis of the network interactions among the various soil microorganisms despite the extremely complex and high diversity of microbial communities in natural soil ecosystems [20]. However, limited studies have applied high-throughput sequencing to analyze the microbial communities in arable soil treated with soil fertilizer/conditioner.

In this study, we report a soil conditioner derived from the dynamic rapid fermentation (DRF) of food waste. We also investigated the effect of the DRF-derived soil conditioner on the quality of OM-impoverished arable soil and strawberry yield. In addition, we studied the changes in the composition of soil microbial community during application of soil conditioner. We also identified the relationship of the composition of the microbial community with the physiochemical parameters after treatment. This study may serve as a reference to direct the production of DRF-derived soil conditioner and the application of this fertilizer in OM-impoverished arable soil.

## Materials and methods

### DRF-derived soil conditioner

Food waste and rice husk were mixed at a ratio of 11: 4 (wet weight) and used as raw materials to produce a soil conditioner (Table 1). The inoculant (~1×10^8^ CFU/mL, 1.25 mL/kg), which contained *Bacillus circulans*, *Bacillus sphaericus*, *Bacillus firmus*, *Bacillus schlegelii*, *Bacillus stearothermophilus*, *Bacillus subtilis*, *Candida tropicali*, and *Lactobacillus delbruckii*, was then added to the mixture. The mixture was loaded into a bioreactor and heated at ~70°C for 9 h, and then heating was stopped to allow the natural cooling of the bioreactor (~9 h). The materials were stirred during the entire process. The materials were subsequently passed through a 5-mm sieve.

**Table 1:** Characteristics of the dynamic rapid fermentation (DRF) -derived soil conditioner.

Parameters	Value
TC/TN	25.40
Moisture content (%)	33.33±0.11
OM (%, DW^a^)	80.00±0.38
pH	7.25±0.14
EC (mS/cm)	2.29±0.12
NH_4_^+^-N (g/kg, DW)	1.42±0.02
NO_3_^-^-N (g/kg, DW)	0.73±0.01

^a^ DW, dry weight; TC, total carbon; TN, total nitrogen; OM, organic matter; EC, electric conductivity.

### Experimental design

The greenhouse experiment was conducted at the Beiwu Field Station located at Chaoyang District, Beijing, China. The soil used in the greenhouse experiment was also collected from Chaoyang, Beijing, China (40.4961°N, 116.4858°E). The collected climate data were consistent with the historical averages for the region, which has a monsoon climate at medium latitude, a relative humidity of 70% to 80%, and an average annual temperature of 11.6°C. The soil in the area is histosol in North China (Table 2) [21]. The soil was unimproved grazing and had not been cultivated and applied with fertilizer for 7 years previously, but mechanical weeding had been occurring during this period. The prepared soil conditioner was applied to the soil in the greenhouse experiment at 320 kg/ha in early February every year.

**Table 2:** Physical and chemical properties of the original soil.

Property	Value
Texture	8.4% clay, 19.1% silt, and 72.5% sand
OM	8.514 ± 0.71 g/kg
pH	7.2 ± 0.2
CEC^a^	13.1 ± 0.6 cmol/kg
Available N	124.453 ± 3.4 mg/kg
Available P	26.49 ± 0.8 mg/kg
Available K	0.228 ± 0.01 g/kg

^a^ CEC, cation exchange capacity.

Before initiating the experiment, the DRF-derived soil conditioner was stabilized in soils for approximately 7 days to 10 days. The soil chemical characteristics were evaluated (Table 2) prior to cultivation. Four replicates (4×3 m^2^ for each plot) were prepared in the experimental blocks. Each plot was ploughed to form a 0.2 m elevated bed covered with black polyethylene inside, with raw spacing of 0.20 m × 0.20 m. Uniform strawberry plants with a 15 mm to 20 mm diameter root section were selected and planted on the elevated bed. The experimental site was equipped with a drip irrigation system, the system was operated for 5 h a day, and every plot was operated with a separate irrigation pipe (T-tape) with water flow of 5 L h^−1^ m^−1^ (1 L water per hour on a single dripper). In each plot, soil samples were randomly collected with a soil sampler (ZH23-300A, China) along the length of a “sample walk” that formed a W shape with a total of 16 sampling points [22]. The soils located at 0 cm to 15 cm were collected at each sampling point. The samples from each plot were mixed thoroughly, and approximately 16 kg of soil was collected. During the 6 years of applying DRF soil conditioner, including the original soil (0Y) and soil samples with soil conditioner (1Y-6Y), a total of 28 samples were collected. These samples were mixed and passed through a 2-mm sieve. The resulting sample was then divided into two parts. One part was air dried and ground to pass through a 0.5-mm sieve for physicochemical analysis, and the other part was stored at −20°C prior to microbiological analysis. The fruit number and weight were measured throughout the experiment to evaluate strawberry yield.

### DNA extraction and 16S rDNA sequencing

Soil samples were washed with buffer solution prior to DNA extraction to avoid the interference of humus. Soil DNA was extracted from each soil sample with the Power Soil DNA Isolation kit (Omega Biotek Inc.) following the manufacturer’s instructions and stored at −20°C prior to further analysis.

The V3 hypervariable region of the 16S rRNA was PCR-amplified from microbial genome DNA which was harvested from soil samples using barcoded fusion primers (forward primer: 338-CCTACGGGAGGCAGCAG-355, reverse primer: 502-ATTACCGCGGCTGCTGG-518). The PCR conditions were as follows: 94°C for 5 min; followed by 25 cycles at 94°C for 30 s, 48°C for 30 s, and 72°C for 30 s; and final extension at 72°C for 10 min. Then, the PCR product was excised from a 2% agarose gel. Sequencing libraries were labeled with different multiplex indexing barcodes using NEB Next Ultra DNA Library Prep Kit for Illumina (New England Biolabs Inc., USA) following the manufacturer’s recommendations. The quality of the libraries was assessed with Qubit 2.0 Fluorometer (Thermo Scientific, Waltham, USA) and Agilent Bioanalyzer 2100 system. The libraries were sequenced on an Illumina MiSeq 2500 platform at the Beijing Center for Physical and Chemical Analysis (China).

### Data analysis using high-throughput sequencing

Software QIIME (Quantitative Insights Into Microbial Ecology) was used to analysis sequence data. Operational taxonomic units (OTUs) were classified at the 97% sequence similarity threshold, and a representative sequence for each OTU was aligned using the Ribosomal Database Project classifier (version 2.2) and GreenGene database [23–24]. Shannon, Chao1, ACE, and Good’s non-parametric coverage estimator indices were calculated using Mothur software to estimate bacterial diversity and richness.

### Analysis of soil properties

Moisture of the samples was determined by the weight loss after drying at 105°C for 24 h. The samples were extracted with deionized water (at 1:10 w/v) to analyze the electric conductivity (EC) using an EC meter [25] and measure soil pH using a pH meter (Mettler Toledo S20K). SOM was determined at 550°C for 2 h in a muffle furnace [26].Total carbon (TC) was measured according to the method of Nelson et al. [27]. Total nitrogen (TN) was determined using a Carlo-Erba NA 1500 C/N analyzer (Carlo Erba Instruments, Milan, Italy). Total phosphorus (TP) was assessed using microwave digestion method [28]. NH_4_^+^-N and NO_3_^−^-N were determined by extracting from the freeze-dried solid samples with 2M KCl, steam distillation, and titration [29].

### Statistical analysis

The means and standard deviations were compared using Duncan test with a significance level of *P*<0.05. Figure processing was generated by Origin 8.0 software (IBM, USA). Canoco for Window (version 4.5, Centre for Biometry, Wageningen, Netherlands) was used for the principal component analysis (PCA) of the relative abundance of the phyla at class level and redundancy analysis (RDA) of the relationship between environmental variables and soil community composition.

## Results and discussion

### Soil characteristics

Continued application of DRF-derived soil conditioner significantly (*P*<0.05) changed soil properties, including SOM, pH, EC, TC, TP, NO_3_^−^-N, and NH_4_^+^-N, and increased strawberry yield, compared with the original soil before soil conditioner added (0Y) (Table 3). Changes in the moisture of the OM-impoverished soil ranged from 7.0% to 20.1% during the six years of treatment. High moisture in soils facilitated the release of compounds accumulated by soil microbes and motivated microbial activity and growth [30]. High EC may result in nitrogen depletion, nutrient reduction, and decrease in strawberry yield [31–32]. The pH level and EC both declined after treatment for six years. The pH level significantly declined from 7.2 to 6.48, and EC significantly changed from 0.062 dS/m to 0.022 dS/m.

**Table 3:** Physical and chemical properties of the soils after applying DRF-derived soil conditioner for 6 years.

DRF application time^1^	TC (g/kg)	TN (g/kg)	TP (g/kg)	Moisture (%)	pH	SOM (g/kg)	EC (dS/m)	NO_3_^−^-N (mg/kg)	NH_4_^+^-N (mg/kg)	Yield (kg ha^−1^)
0Y(CK)	8.396 (0.034)a	0.78 (0.128)a	0.41 (0.298)a	7.0 (0.020)a	7.20 (0.072)b	8.514 (0.006)a	0.062 (0.003)e	17.541 (2.077)a	97.222 (3.274)c	1836.58 (36.621)a
1Y	13.872 (0.012)a	1.38 (0.226)a	0.55 (0.244)a	12.6 (0.025)b	7.14 (0.161)b	11.609 (0.013)ab	0.041 (0.002)cd	22.117 (0.782)b	67.652 (4.581)a	2157.34 (43.296)ab
2Y	14.393 (0.018)ab	1.40 (0.110)a	0.58 (0.149)a	11.7 (0.031)b	7.21 (0.115)b	11.806 (0.007)b	0.031 (0.011)abc	26.693 (3.573)c	99.624 (7.065)c	2198.17 (52.670)ab
3Y	16.288 (0.038)b	1.41 (0.373)a	0.57 (0.311)a	10.9 (0.013)b	7.11 (0.026)a	12.460 (0.005)b	0.027 (0.004)ab	23.642 (2.110)bc	86.747 (5.637)b	2206.15 (45.113)ab
4Y	15.935 (0.044)b	1.81 (0.483)ab	0.80 (0.346)a	14.8 (0.021)bc	6.88 (0.290)a	12.113 (0.004)b	0.033 (0.002)bcd	38.896 (2.277)d	97.445 (0.877)c	2354.88 (51.710)b
5Y	18.240 (0.156)c	2.66 (0.164)bc	1.51 (0.200)b	17.8 (0.012)cd	6.76 (0.075)a	13.704 (0.012)b	0.042 (0.002)d	26.646 (1.141)bc	89.453 (1.462)b	2384.76 (54.082)b
6Y	19.012 (0.070)c	3.03 (1.255)c	1.63 (0.269)b	20.1 (0.019)d	6.48 (0.084)a	13.806 (0.004)b	0.022 (0.009)a	28.631 (2.560)bc	93.178 (1.662)bc	2392.49 (46.221)b
ANOVA P-values^2^	<0.001	0.002	<0.001	<0.001	<0.001	<0.001	<0.001	<0.001	<0.001	<0.001

^1^DRF application time: 0Y (without DRF-derived soil conditioner), 1Y–6Y (fertilized DRF-derived soil conditioner for 1 year to 6 years). TC, total carbon; TN, total nitrogen; TP, total phosphorous; SOM, soil organic matter; EC, electric conductivity.

^2^Values in bracket are mean±standard deviation (N = 3). Values within the same column followed by different letters indicate significant difference.

The SOM improved 5.3 g/kg after applying DRF-derived soil conditioner for 6 years (Table 3), the SOM content appeared an increasing change except for the fourth year. When the conditioner was applied to the soil, SOM was decomposed and utilized by the soil microbes. As the cumulative rate of SOM was greater than the consumption rate, the SOM content increased gradually. TC, TN, and TP increased by 1.40, 2.25, and 1.22 g/kg, respectively, after six years of treatment. NO_3_^−^-N also increased by 63.22% compared with that in 0Y, because the DRF-derived soil conditioner was high TC/TN. Physical and chemical properties of the soils were mostly changed because of the increased functional microbes in soils were involved in the carbon, nitrogen, and phosphorous fixation.

### Diversity of microbial communities in soil

**Fig 1:**
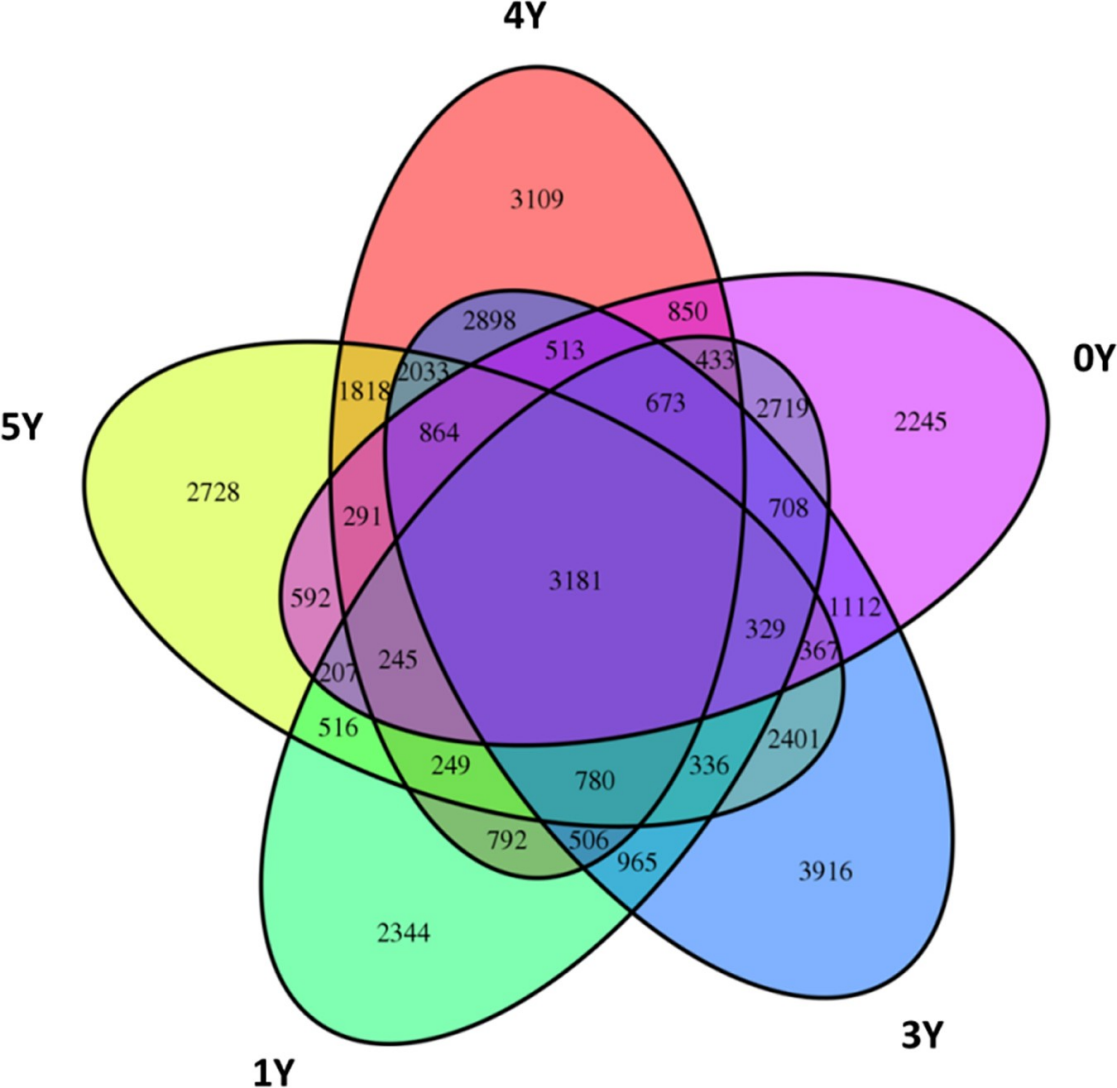
Venn diagram presents the total number of operational taxonomic units (OTUs) shared by soils treated for 0 (pink), 1 (green), 3 (blue), 4 (red) and 5 (yellow) years. The numbers in one circle denote year-specific OTUs, and numbers in two or more intersecting circles denote overlapped OTUs.

The number of OTUs in the soil samples ranged from 10298 to 21582 (Table 4). The soil sample collected on the sixth year (6Y) possessed the lowest number of total OTUs, while those on the third year (3Y) had the highest value. The Venn diagram in Fig 1 reflects 2.9% of OTUs were shared in all samples. The majority of the shared OTUs belonged to Firmicutes, Proteobacteria, and Actinobacteria (Table 5). After six years of continuous application of DRF-derived soil conditioner, the Venn diagram indicated good homology for the bacterial communities, which could be ascribed to the same source of the soils. Significant differences were observed in valid sequences, OTUs, microbial richness for Chao1 and ACE, but not in Good’s coverage and Shannon. Good’s coverage values ranged from 0.92 to 0.95 at 97% similarity cutoff (Table 4), indicating that the current numbers of sequence reads were sufficient to capture the bacterial diversity in these soils.

**Table 4:** Microbial diversity indices of soils after application of DRF-derived soil conditioner.

Application time	Valid sequences	OTUs^1^	Coverage (%)^2^	Chao1	ACE	Shannon
0Y(CK)	138001 (921)b	15329 (384)b	0.944 (0.020)a	24865.12 (128)c	27917.78 (798)b	10.137 (0.076)ab
1Y	152467 (9129)b	14983 (505)b	0.951 (0.030)a	23783.14 (555)bc	26746.18 (232)b	9.510 (0.432)a
2Y	82916 (104)a	13035 (1705)ab	0.919 (0.002)a	21384.82 (153)ab	24337.25 (1633)b	10.766 (0.007)b
3Y	201400 (7779)d	21582 (82)c	0.947 (0.021)a	34173.36 (640)e	38874.37 (760)e	10.647 (0.072)b
4Y	180293 (882)c	19235 (161)c	0.946 (0.023)a	30788.37 (2694)e	35222.47 (399)d	10.367 (0.163)ab
5Y	142333 (8725)b	16937 (846)b	0.937 (0.014)a	28606.11 (554)d	32518.65 (122)c	10.125 (0.048)ab
6Y	76432 (668)a	10298 (2027)a	0.925 (0.036)a	19203.08 (1774)a	21375.21 (397)a	10.032 (0.110)ab
ANOVA P-values	P<0.001	P = 0.001	P = 0.786	P<0.001	P<0.001	P = 0.179

^1^OTUs: operational taxonomic units (97% similarity).

^2^Coverage: Good’s non- parametric coverage estimator.

**Table 5:** Phylum distribution of microbial community from soils after application of DRF-derived soil conditioner.

Order	Phylum	Relative abundance of community (%)						
		0Y (CK)	1Y	2Y	3Y	4Y	5Y	6Y
1	Firmicutes	3.74	4.03	3.86	11.81	16.18	20.66	19.61
2	Proteobacteria	35.27	40.10	40.41	34.78	29.60	23.28	31.43
3	Actinobacteria	22.22	17.36	11.19	14.62	15.71	11.40	12.86
4	Acidobacteria	12.86	16.03	10.49	12.52	12.50	14.51	6.98
5	Bacteroidetes	6.47	3.67	7.55	4.70	6.01	3.44	8.39
6	Gemmatimonadetes	2.63	2.58	6.20	5.39	5.13	4.48	4.54
7	Chloroflexi	5.84	3.29	5.02	4.85	4.51	4.72	6.65
8	Verrucomicrobia	2.05	1.50	0.15	0.20	0.25	0.30	0.72
9	Nitrospirae	1.10	1.20	1.61	1.56	2.07	1.54	1.61
10	TM7	1.20	1.00	1.66	1.26	1.00	0.44	1.09
11	Planctomycetes	0.55	0.84	1.43	1.08	0.57	1.04	0.38
12	Other	5.07	7.29	9.26	5.67	4.71	12.43	3.41

ACE, Chao1, coverage, and Shannon index showed that the DRF-derived soil conditioner affected the richness and diversity of the soil bacterial community to a certain extent. Specially, on the second year of application, the lower ACE and Chao1 values were contrary to the highest Shannon index. This result indicated that microbial richness decreased, but microbial diversity considerably increased [33] in the treated soils compared with unfertilized soil. Shannon’s index slightly decreased with continuous treatment. This phenomenon may be ascribed to the fact that the rich OM in the DRF-derived soil conditioner facilitated the reproduction of certain microbial groups. However, several other microbial groups exerted weaker competiveness, resulting in a decrease in the bacterial diversity in the soils.

### Taxa assignments

The relative abundances of the top 12 phyla of soil microbial community are shown in Table 5. Proteobacteria, Actinobacteria, Acidobacteria, and Firmicutes were the dominant phyla, occupying 65.95%–77.52% of the bacterial sequences obtained from the soil samples.

The sequence affiliated with the phylum Proteobacteria occupied the highest proportions (23.28%–40.41%), and the relative abundances of Nitrospirae appeared significantly enhanced and reached its peak (2.07%) on the fourth year of treatment. The presences of Proteobacteria and Nitrospirae have remarkable relationship with the changes in TN, NO_3_^−^-N and NH_4_^+^-N in the soils after treatment. This phenomenon could have been caused by the possible involvement of a large amount of Proteobacteria (such as Hydrogenophaga and Acidovorax) and increasingly relative abundant of Nitrospirae in the nitrogen transformation and cycle through metabolism [34–35], influencing the physicochemical properties of soils.

Bacteroidetes and Chloroflexi occupied 6.47% and 5.84% of the total bacteria in the original soil (0Y), respectively. These bacteria were enriched and reached their peaks of 8.39% and 6.65% on the sixth year of application. Actinobacteria and Acidobacteria generally decreased in all soil samples compared with 0Y. This reduction was possibly due to the relatively ineffective competitiveness of these bacteria under high nutrient conditions against other bacteria [36].

**Fig 2:**
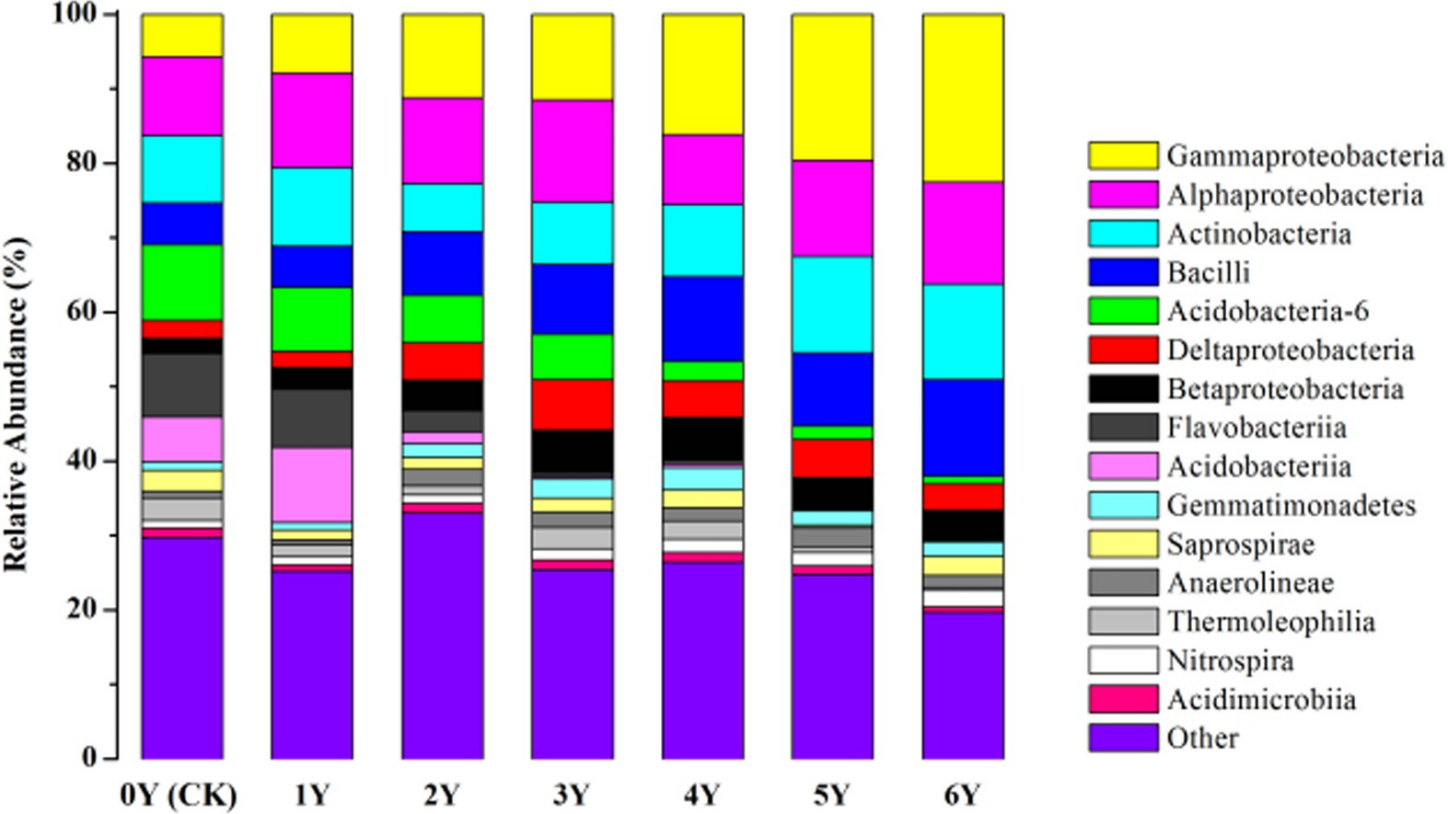
Relative abundance of the dominant bacterial class across soils with or without application of DRF-derived soil conditioner from 0 year to 6 years. 0Y, control sample. 1Y–6Y, soils treated with DRF-derived soil conditioner for 1 year to 6 years.

A total of 15 main classes were determined (Fig 2). Bacilli and Nitrospira significantly increased in the treated soils compared with those in 0Y, and reached their peaks at 12.99% and 2.33% on the sixth year of application. Bacilli are the major component of the inoculant in the DRF-derived soil conditioner because of its wide temperature-tolerance limit (~70°C from the heating bioreactor) [37]. After soil treatment, Bacilli remained dominant because of its good adaptability. Bacilli are best known for its degradation of macromolecules, such as cellulose starch and protein, into small molecules (Manz et al., 1996; 35; Kumar &Purohit 2012; 36) [38–39], which are beneficial to the fermentation of food wastes. In addition, Bacilli in the soils play a dominant role on nitrogen fixation [35]. The increased distribution of Bacilli and Nitrospira increased NO_3_^−^-N in the soils after treatment (Table 3).

Members of Actinobacteria are widely distributed terrestrially [40]. These bacteria are frequently involved in the turnover of OM and played an important role in the degradation of recalcitrant compounds [41]. The soil was OM-impoverished before application of DRF-derived soil conditioner. The relative abundance of Actinobacteria greatly improved after the addition of OM-rich DRF-derived soil conditioner (Fig 2), indicating that Actinobacteria is involved in the transformation of OM in the DRF-derived soil conditioner into SOM. Additionally, the increase of the NO_3_^−^-Nin the soils and strawberry yields may be ascribed to the presence of Actinobacteria because of its impact on nitrogen fixation associated with non-leguminous plants [42].

The relative abundances of four subdivisions of Proteobacteria, namely, Gammaproteobacteria, Alphaproteobacteria, Betaproteobacteria, and Deltaproteobacteria, were the major community components in the soils after six years of treatment (Fig 2). This phenomenon may be ascribed to the fact that the inoculant in the DRF-derived soil conditioner can produce several enzymes, such as protease, lipase, amylase, and chitinase. This process is conducive to the degradation of macromolecular protein and fat into small molecules, such as polypeptide and oligosaccharides, which can be utilized by Betaproteobacteria for growth [43–44]. Betaproteobacteria and Deltaproteobacteria were the main subdivisions of Proteobacteria from the second to the fourth years of treatment. Alphaproteobacteria and Gammaproteobacteria were dominant in the soil microbial community in the last two years of application, with relative abundances of 13.62% and 22.55%, respectively. Gammaproteobacteria were more abundant in long-term fertilized soils compared with 0Y [45–46]. Several members of Gammaproteobacteria accounted for 40%–70% of CO_2_ fixation using sulfur as the electron donor [47]. This phenomenon may account for the increased TC in the soils during treatment.

**Fig 3:**
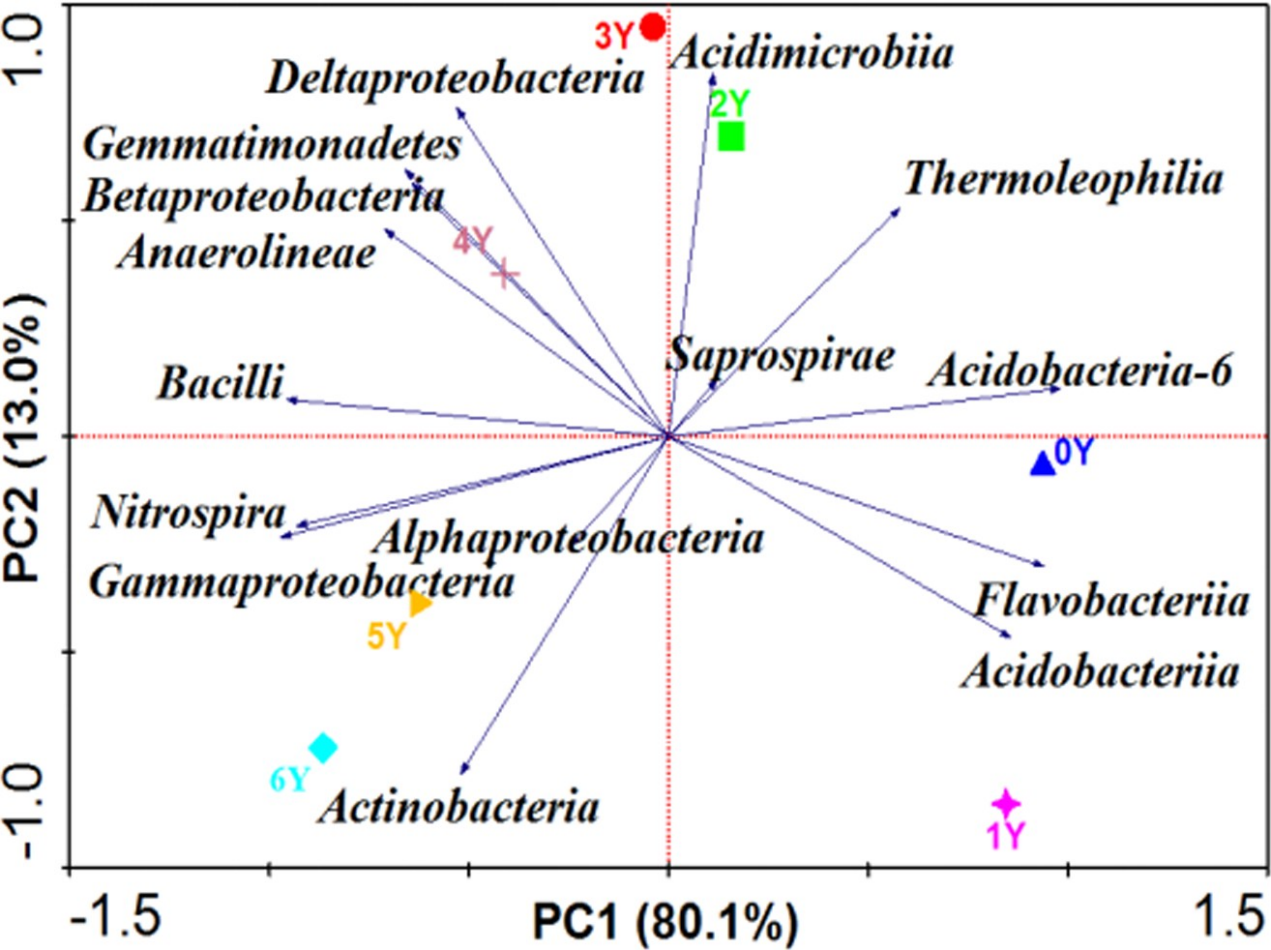
Principal component analysis of class abundance in soils under 0–6 years of long-term application of DRF-derived soil conditioner.

Classes which occupied the majority of the bacterial sequences were analyzed using PCA (Fig 3). PCA results demonstrated that the microbial communities could be separated using the class abundance data set, indicating that applying DRF-derived soil conditioner for six years significantly changed the distribution of major soil microbial communities. Specifically, Acidobacteria-6, Flavobacteriia, Acidobacteriia, Saprospirae, Thermoleophilia, and Acidimicrobiia were enriched in the soil microbial community after application of DRF-derived soil conditioner for two years. Bacilli, Gemmatimonadetes, Betaproteobacteria, Deltaproteobacteria, and Anaerolineae were abundant after application of DRF-derived soil conditioner for 3–4years.Alphaproteobacteria, Actinobacteria, Nitrospira, and Gammaproteobacteria were more abundant in 5Y and 6Y.This result suggested that the microbial community was largely influenced by the application of DRF-derived soil conditioner.

The major genera significantly changed during application of DRF-derived soil conditioner (Table 6). The dominating genera prior to treatment were Flavobacterium and Shewanella, which were replaced by Bacillus and Rhodoplanes after six years of treatment. The corresponding proportions of Bacillus and Rhodoplanes were 4.36 and 1.66 times higher than those in 0Y. These changes might have been caused by the high ability of Bacillus to degrade cellulose and its wide temperature-tolerance limit in the environment [37]. After treatment for six years, Flavobacterium and Shewanella disappeared, but Streptococcus, Acinetobacter, and Lactobacillus appeared in the top 10 most abundant genera at the sixth year. This phenomenon suggested that application of DRF-derived soil conditioner has significantly changed the distribution of the major functional microbial community.

**Table 6:** contribution of the top 10 most abundant genera detected in 6 years soils after application of DRF-derived soil conditioner.

	Genus/contribution to total number of sequences (%)													
NO.	0Y (CK)		1Y		2Y		3Y		4Y		5Y		6Y	
1	Flavobacterium	1.4	Shewanella	1.7	Shewanella	2.17	Bacillus	1.71	Streptococcus	3.86	Lactobacillus	2.74	Bacillus	4.88
2	Shewanella	1.24	Rhodoplanes	1.25	Rhodoplanes	0.86	Streptococcus	1.55	Rhodoplanes	1.83	Bacillus	2.4	Rhodoplanes	1.69
3	Bacillus	1.12	Bacillus	0.98	Bacillus	0.83	Rhodoplanes	1.36	Bacillus	1.21	Rhodoplanes	1.89	Nitrospira	1.44
4	Rhodoplanes	1.02	Flavobacterium	0.71	Nitrospira	0.73	Lactobacillus	0.98	Nitrospira	0.99	Sporosarcina	1.28	Streptococcus	1.2
5	Nitrospira	0.41	Nitrospira	0.58	Streptococcus	0.49	Nitrospira	0.87	Prevotella	0.8	Streptococcus	1.18	Balneimonas	0.71
6	Balneimonas	0.39	Balneimonas	0.57	Flavobacterium	0.29	Balneimonas	0.45	Balneimonas	0.49	Nitrospira	0.86	Paenibacillus	0.68
7	Rhodococcus	0.35	Rhodococcus	0.32	Prevotella	0.27	Flavobacterium	0.39	Lactobacillus	0.44	Paenibacillus	0.61	Acinetobacter	0.46
8	Pseudomonas	0.28	Pseudomonas	0.25	Balneimonas	0.22	Paenibacillus	0.25	Asticcacaulis	0.28	Pseudomonas	0.25	Lactobacillus	0.29
9	Paenibacillus	0.19	Paenibacillus	0.18	Rhodococcus	0.13	Pseudomonas	0.23	Shewanella	0.27	Prevotella	0.2	Rhodococcus	0.25
10	Leucobacter	0.12	Leucobacter	0.09	Paenibacillus	0.11	Asticcacaulis	0.21	Flavobacterium	0.26	Flavobacterium	0.19	Leucobacter	0.24

### Correlations of microbial communities with edaphic variables

**Fig 4:**
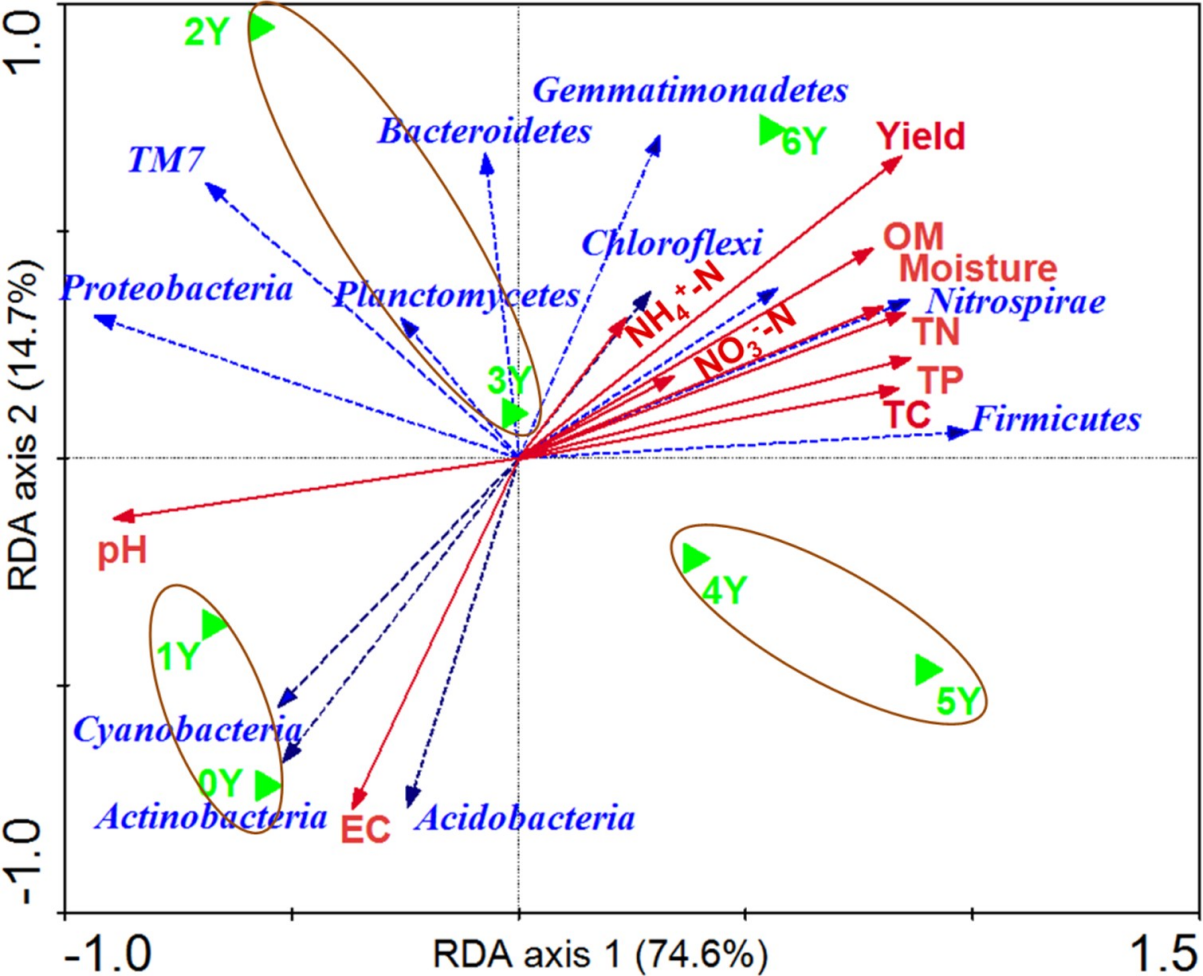
Redundancy analysis of related DRF soil conditioner sequence patterns with edaphic variables for six years. The bacterial communities and samples are indicated in blue and green colors, respectively. Soil factors indicated in red text include total carbon (TC), total nitrogen (TN), total phosphorus (TP), NH_4_^+^-N, NO_3_^-^-N, organic matter (OM), soil electric conductivity (EC), moisture, and yield.

According to the RDA, soil pH and TP were identified as more important contributors to the variation in bacterial communities (Fig 4). These values were followed by TC, TN, moisture, OM, EC, NH_4_^+^-N, NO_3_^−^-N. Based on this model, 89.3% of the total variance was explained by the first two constrained axes of the RDA. The bacterial communities were divided into three groups, namely, 2Y and 3Y, 4Y and 5Y, and they separately from 0Y and 1Y soil samples.

Most of the abundant phyla (relative abundance >0.5%) were significantly correlated with the environmental factors (Fig 4). The relative abundances of Gemmatimonadetes, Chloroflexi, Verrucomicrobia, Nitrospirae and Firmicutes were significantly correlated with soil TC, TN, TP, NO_3_^−^-N, NH_4_^+^-N, OM, and moisture. These bacteria were all distributed in the sixth year of application of DRF-derived soil conditioner. That may be the reason why the relative abundance of these microbial communities was significantly enriched along with the increase of the above mentioned soil properties. The same result was observed in the research of Zhou [48] and Nemergut [49]. While Proteobacteria, TM7, and Planctomycetes showed no significant correlation with any soil property, indicating their greater resistance and resilience to disturbance or environmental changes.

Cyanobacteria, Actinobacteria, and Acidobacteria were correlated with pH and EC. These bacteria were mainly distributed in 0Y and 1Y. Many studies have suggested that the relative abundance of Actinobacteria had a strong positive [50–51] or negative [3] correlation with pH. However, reports also show that Actinobacteria and Acidobacteria had no significant correlation with any of the tested soil characteristics [52]. A recent study showed that pH strongly influence the composition of soil microbial communities, with soil pH being a good predictor of bacterial community composition [48]. However, Tian et al. [52] figured out that it was inexact when they distinguished what was more effective in shaping bacterial communities between pH and other soil characteristics, because pH was significantly correlated with only several of the abundant phyla. Generally, our results showed that soil OM, nutrient status, and pH were the key impact factors of the soil ecosystems. The presence of DRF-derived soil conditioner greatly influenced the soil physicochemical properties, altering the distribution and function of microbial communities.

### Strawberry yields

Strawberry yields constantly increased during treatment. On the sixth year, strawberry yields increased by 555.91 kg/ha compared with that without applying soil conditioner (Table 3). This phenomenon was greatly associated with pH changes, because the optimum pH for strawberry growth is 5.5–6.5 [31]. Plant productivity is more sensitive to soil pH and nutrient [53], because it directly influences the uptake of mineral nutrients by the soil microorganism. Reduced availability of micronutrients may occur at high pH levels [54]. Increase in strawberry yields was not significant from the second year to the third year of application of DRF-derived soil conditioner but became significant after four years of treatment. This phenomenon can ascribed to the high accumulation of TN, NH_4_^+^-N, and NO_3_^−^-N after four years of application of DRF-derived soil conditioner.

## Conclusions

The effect of long-term application of DRF-derived soil conditioner on OM-impoverished arable soil was evaluated in this study. Significantly increase in TC, TN, and TP, 1.6-fold accumulation of SOM, and reduced pH and EC were observed during the six years of application of DRF-derived soil conditioner. Analysis of the soil microbial community structure suggested that the DRF-derived soil conditioner greatly influenced microbial activity and soil characteristics. The distributions of the major functional soil microbial communities, especially those involved in carbon sequestration and nitrogen fixation, were significantly changed. The soil conditioner derived from DRF of food wastes presented good potential to improve the quality and optimize the microbial community of OM-impoverished soils and enhance strawberry yields.
